# Association between alcohol dietary pattern and prevalence of dyslipidaemia: WASEDA’S Health Study

**DOI:** 10.1017/S0007114521002671

**Published:** 2022-06-14

**Authors:** Kumpei Tanisawa, Tomoko Ito, Ryoko Kawakami, Chiyoko Usui, Takuji Kawamura, Katsuhiko Suzuki, Shizuo Sakamoto, Kaori Ishii, Isao Muraoka, Koichiro Oka, Mitsuru Higuchi

**Affiliations:** 1 Faculty of Sport Sciences, Waseda University, 2-579-15 Mikajima, Tokorozawa, Saitama 359-1192, Japan; 2 Waseda Institute for Sport Sciences, 2-579-15 Mikajima, Tokorozawa, Saitama 359-1192, Japan

**Keywords:** Diet, Drinking behaviour, Hyperlipidaemia, LDL-cholesterol, TAG, Japan

## Abstract

The association between a dietary pattern characterised by high alcohol intake and dyslipidaemia has not been fully investigated. Therefore, the present study aimed to investigate the association between alcohol dietary patterns and the prevalence of dyslipidaemia and its components. This cross-sectional study enrolled 2171 men and women aged ≥40 years who were alumni of a Japanese university. To identify dietary patterns, a principal component analysis was performed based on the energy-adjusted food intake estimated by a brief-type self-administered diet history questionnaire. Three dietary patterns were identified, the second of which was named the alcohol dietary pattern and was characterised by a high intake of alcoholic beverages, liver, chicken and fish. This alcohol dietary pattern was associated with reduced LDL-cholesterol levels. The fully adjusted OR (95 % CI) of high LDL-cholesterol for the lowest through highest quartile of alcohol dietary pattern score were 1·00 (reference), 0·83 (0·64, 1·08), 0·84 (0·64, 1·10) and 0·68 (0·49, 0·94), respectively. Subgroup analysis showed that the alcohol dietary pattern was inversely associated with the prevalence of dyslipidaemia in women, whereas it was positively associated with high TAG levels in men. In conclusion, the alcohol dietary pattern, characterised by a high intake of alcoholic beverages, liver, chicken and fish, was associated with the prevalence of dyslipidaemia and its components. This finding provides useful information for the prevention and treatment of dyslipidaemia by modifying the diet.

Many epidemiological studies have demonstrated that high alcohol intake is associated with an increased risk of mortality^(1,2)^, various types of cancer^(3,4)^ and CVD^(2)^. Although a curvilinear dose–response relationship between alcohol intake and the risk of chronic diseases and mortality has often been observed, a meta-analysis of cohort studies has demonstrated a J-shaped association between alcohol intake and the incidence of CHD^(1,4)^. A reduction in the risk of CHD due to moderate alcohol intake is plausible, due to an improvement in the blood lipid profile. A meta-analysis of intervention studies has indicated that moderate alcohol intake significantly increases circulating HDL-cholesterol levels^(5,6)^, which has been proposed to have anti-atherogenic properties^(7)^. Furthermore, several studies have reported that alcohol intake was inversely associated with circulating LDL-cholesterol levels^(8–10)^, which is a major cause of CHD, although other studies have conversely reported that heavy alcohol intake was associated with increased LDL-cholesterol levels^(11,12)^. Alcohol intake is also associated with circulating TAG levels, and J-shaped or U-shaped associations have been observed in several studies^(13,14)^.

Although several studies have shown the association between alcohol intake and blood lipid profile as described above, the association of a dietary pattern characterised by high alcohol intake with blood lipid or dyslipidaemia has not been fully investigated. Alcohol intake is potentially one of the most important determinants of dietary habits, a fact which is supported by a genetic association study showing that alcohol consumption-associated genetic loci are strongly associated with dietary habits^(15)^. A diet consumed with high alcohol intake may not only affect lipid metabolism but also confound the association between alcohol intake and blood lipid levels. Therefore, studies on dietary patterns specific to alcohol drinkers will help to better understand the relationship between alcohol intake and lipid metabolism. Several studies conducting dietary pattern analysis by principal component analysis have identified the ‘alcohol dietary pattern’ as the major dietary pattern. Interestingly, the factor loadings characterising alcohol dietary patterns differed among the studies, and various patterns such as ‘red meat and alcohol’^(16,17)^, ‘snacks and alcohol’^(18,19)^ and ‘noodles and alcohol’^(20,21)^ have been proposed. Therefore, there is a possibility that a dietary pattern specific to alcohol drinkers in each study has a different impact on the blood lipid profile. We previously identified a dietary pattern characterised by a high intake of alcoholic beverages and seafood among alumni of a Japanese university^(22)^. Because the consumption of fish, fish oil and alcohol intake itself has been reported to improve the blood lipid profile, the alcohol dietary pattern identified in Japan may be inversely associated with the prevalence of dyslipidaemia, and investigating it will provide useful information for the prevention and treatment of dyslipidaemia by modifying the diet. Therefore, we performed a cross-sectional study consisting of middle-aged and elderly individuals who are alumni of a Japanese university to examine the association between the alcohol dietary pattern and the prevalence of dyslipidaemia and its components.

## Experimental methods

### Participants

In this cross-sectional study, we used baseline survey data from the Waseda Alumni’s Sports, Exercise, Daily Activity, Sedentariness, and Health Study (WASEDA’S Health Study), which is a prospective cohort study aimed at examining the relationship between physical activity, exercise, sedentary behaviour, health outcomes and various correlates among the alumni of Waseda University and their spouses aged ≥40 years. The WASEDA’S Health Study consists of four cohorts (cohorts A–D) with different measurement items, and the participants selected one of the four cohorts when they registered for the study. The present study comprised a total 2544 individuals (men: *n* 1614, women: *n* 930) who participated in the baseline survey of cohort C or D between March 2015 and February 2020. We excluded participants based on the following criteria: (1) a history of heart diseases (*n* 196); (2) incomplete web-based questionnaires (*n* 93); (3) incomplete dietary survey (*n* 19); (4) extreme self-reported energy intake (<600 kcal/d or ≥4000 kcal/d) (*n* 10); (5) extreme self-reported physical activity (*n* 26); (6) consumption of breakfast before blood sampling (*n* 22) and (7) lack of blood biochemical parameters (*n* 7). Based on the above criteria, 2171 individuals (men, *n* 1354, mean age 55·5 (sd 10·1) years; women, *n* 817, mean age 51·5 (sd 8·1) years) were included in the analysis. All participants provided written informed consent before enrolment in the study, which was approved by the Ethical Committee of Waseda University (reference number. 2014-095, 2014-G002, 2018-320 and 2018-G001). The study was conducted in accordance with the principles of the Declaration of Helsinki.

### Anthropometry

The participants visited the laboratory between 08.30 and 10.30 hours, and all measurements were conducted by trained investigators. Height was measured using a stadiometer (YHS-200D, YAGAMI Inc.). Body weight was measured using an electronic scale (MC-980A, Tanita Corp.), with the participants wearing light clothing and no shoes. BMI was calculated as body weight (kg) divided by the square of the body height (m). Waist circumference was measured at the umbilical region with an inelastic measuring tape at the end of normal expiration to the nearest 0·1 cm.

### Blood sampling and analysis

Venous blood samples were collected by venepuncture after at least 12 h of overnight fasting. Blood samples were collected into serum separation tubes or EDTA-containing tubes and subsequently centrifuged at 3000 rpm at 4°C for 10 min using a centrifuge (Model 5911, Kubota). The serum levels of aspartate aminotransferase (AST), alanine aminotransferase (ALT) and *γ*-glutamyl transferase, and the concentrations of LDL-cholesterol, HDL-cholesterol, TAG, fasting insulin and plasma concentrations of fasting glucose were determined at BML, Inc.. The ALT:AST ratio was calculated as an index of alcoholic liver disease. The hepatic steatosis index^(23)^ was calculated as an index of non-alcoholic fatty liver disease from the ALT, AST, BMI, sex and the presence of diabetes mellitus, using the following formula:

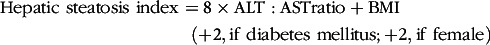




Homoeostasis model assessment of insulin resistance (HOMA-IR) was calculated from the fasting concentrations of plasma glucose and serum insulin as follows:

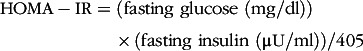




### Dietary assessment

Dietary intake was assessed using a validated brief-type self-administered diet history questionnaire (BDHQ) in the preceding month, as described previously^(22,24)^. We carefully checked all the answered BDHQ to avoid the effect of misreporting. The BDHQ is a four-page questionnaire that takes about 15 min to complete. The dietary intake of fifty-eight food and beverage items, energy and selected nutrients were estimated using an *ad hoc* computer algorithm for the BDHQ, based on the Standard Tables of Food Composition in Japan^(25)^. The validity of the dietary intake data (energy, nutrients and foods) assessed by the BDHQ was confirmed using 16-d semi-weighted dietary records as the gold standard^(26,27)^.

### Other variables

Brachial systolic blood pressure and diastolic blood pressure were measured using the oscillometric method (HEM-7122; OMRON, Inc.), with participants at rest in a sitting position. Physical activity, marital status (yes or no), educational status (junior high/high school, junior college, technical college, college diploma), household income (<3 000 000 JPY, 3 000 000–5 000 000 JPY, 5 000 000–7 000 000 JPY, 7 000 000–10 000 000 JPY, >10 000  000 JPY), smoking status (current smoker, former smoker, non-smoker), use of cholesterol-lowering, TAG-lowering, antihypertensive and diabetes drugs (yes, no) were assessed via a web-based questionnaire survey. Moderate-to-vigorous physical activity (MVPA) was assessed using the Global Physical Activity Questionnaire^(28)^ to quantify the physical activity level, and time spent in total MVPA (min/d) was calculated.

### Definition of dyslipidaemia

Dyslipidaemia was defined as having at least one of the following components: high LDL-cholesterol levels (high LDL-cholesterol: fasting LDL-cholesterol level ≥140 mg/dl or use of cholesterol-lowering drugs), low HDL-cholesterol levels (low HDL-cholesterol, fasting HDL-cholesterol level <40 mg/dl for men; <50 mg/dl for women) and high TAG levels (high TAG: fasting TAG level ≥150 mg/dl or use of TAG-lowering drugs) according to the diagnostic criteria for assessing dyslipidaemia in Japan^(29)^. Because there is a sex difference in HDL-cholesterol of approximately 10 mg/dl, and several guidelines have defined low HDL-cholesterol as <50 mg/dl in women^(30,31)^, we adopted different values to define low HDL-cholesterol in men and women.

### Statistical analysis

To identify dietary patterns, we performed principal component analysis based on energy-adjusted food intake using a density method of fifty-two food and beverage items, as described previously^(22,24)^. We retained three factors based on their eigenvalues (>1), the slope of the scree plot and the interpretability of the factors. The differences in the characteristics across the quartiles of the alcohol dietary pattern scores were assessed by linear regression analysis (for continuous variables) and the Mantel–Haenszel *χ* ^2^ square test (for categorical variables). To evaluate the associations between each dietary pattern and the prevalence of dyslipidaemia and its components, we performed a logistic regression analysis and calculated the multivariate-adjusted OR and 95 % CI for the prevalence of dyslipidaemia, high LDL-cholesterol, low HDL-cholesterol and high TAG according to the quartile of each dietary pattern score, with the lowest quartile as the reference category. Since variables such as basic biological, socio-economic, medication and lifestyle habits were potentially related to both dietary patterns and blood lipid levels, model 1 was adjusted for age, sex, marital status, educational status, household income, use of antihypertensive drugs, use of diabetes drugs, smoking status, MVPA and energy intake. Model 2 was additionally adjusted for waist circumference and HOMA-IR to evaluate whether the observed association was mediated by abdominal obesity and insulin resistance. Model 3 was further adjusted for alcohol intake to evaluate the independent association between each dietary pattern and the prevalence of dyslipidaemia and its components. Sensitivity analysis excluding participants who took any medication was performed to evaluate potential bias associated with including those on medication. We additionally performed a subgroup analysis by sex to evaluate whether the association between the alcohol dietary pattern and the prevalence of dyslipidaemia and its components differed by sex. We added an interaction term (sex × dietary pattern (quartile)) to the model to test the significance of the interaction. The level of statistical significance was set at *P* < 0·05. All statistical analyses were performed using SPSS Statistics (version 25.0; SPSS, Inc.).

## Results

### Dietary pattern

We identified three dietary patterns using principal component analysis (Table 1). The first factor was named the healthy dietary pattern because it was characterised by a high intake of vegetables, fruits, soya products and fish. The second factor was characterised as a high intake of alcoholic beverages, liver, chicken and fish and was named the alcohol dietary pattern. The alcohol dietary pattern identified in the present study was similar to the ‘seafood and alcohol’ dietary pattern that we previously identified in the same cohort^(22)^. However, because the factor loadings of several items of seafood for the second factor were smaller than those of liver and chicken in the present study, we did not name the second factor as ‘seafood and alcohol’. The third factor was characterised by a high intake of pickled vegetables, noodles and fish, as well as low intake of animal meat (beef, pork and chicken) and was named the traditional Japanese dietary pattern. The first to third dietary patterns explained 19·1 % of the variance in food intake.

**Table 1.** Factor loading matrix for each dietary pattern identified by principal component analysis

Food groups	Healthy dietary pattern	Alcohol dietary pattern	Traditional Japanese dietary pattern
Beer	–0·278	0·429	
Shochu	–0·222	0·429	
Sake		0·356	
Wine		0·327	
Liver		0·314	
Squid/octopus/shrimps/shellfish	0·182	0·280	0·218
Whisky	–0·150	0·276	
Small fish with bone	0·314	0·263	0·212
Chicken	0·191	0·255	–0·394
Japanese noodles		0·245	0·354
Oily fish	0·292	0·223	0·350
Lean fish	0·305	0·202	0·227
Dried fish/salted fish	0·227	0·176	0·329
Pickled green leaves vegetable	0·203	0·169	0·348
Pickled other vegetables	0·159		0·408
Buckwheat noodles			0·310
Green tea	0·156		0·280
Natto*	0·246		0·180
100 % fruit and vegetable juice			0·170
Chinese noodle	–0·320		0·163
Low-fat milk			
Seaweeds	0·547		
Pasta	–0·184		
Tomatoes	0·430		
Black tea/oolong tea			
Miso soup			
Canned tuna			
Potatoes	0·350		
Cola drink/soft drink	–0·180		
Lettuces/cabbage (raw)	0·443		
Japanese radish/turnip	0·642		
Tofu/atsuage†	0·448		
Rice	–0·332		
Mushrooms	0·651		
Hum/sausage/bacon			
Coffee			
Mayonnaise/dressing			
Carrots/pumpkin	0·706		–0·158
Egg	0·265		–0·162
Green leaves vegetable	0·701		–0·167
Cabbage/Chinese cabbage	0·659		–0·170
Other root vegetables	0·684		–0·215
Pork/beef			–0·427
Milk/yogurt	0·150	–0·215	
Persimmons/strawberries/kiwifruit	0·334	–0·223	0·279
Ice cream		–0·245	
Citrus fruit	0·271	–0·246	0·293
Other fruit	0·387	–0·257	0·245
Japanese confectioneries		–0·416	0·256
Rice crackers/rice cake/okonomiyak‡		–0·430	
Bread		–0·507	
Western-type confectioneries		–0·585	
Variance explained (%)	10·2	5·0	3·9

A factor loading less than ± 0·15 is not shown.

* Fermented soyabeans.

† Deep-fried tofu.

‡ Savoury pancake with various ingredients (meat, fish and vegetable).

### Characteristics of participants according to the quartile of alcohol dietary pattern score

Because this study focused on the alcohol dietary pattern, we compared the characteristics of participants across the quartiles of alcohol dietary pattern scores (Table 2). Compared with individuals with lower alcohol dietary pattern scores, those with higher scores were more likely to be men, more likely to report higher educational status, higher household income, higher use of TAG-lowering and antihypertensive drugs and were also more likely to be current or former smokers. The alcohol dietary pattern was positively associated with height, body weight, BMI, waist circumference, systolic blood pressure, diastolic blood pressure, AST, ALT, *γ*-glutamyl transferase, hepatic steatosis index, TAG, fasting glucose, HOMA-IR and MVPA, whereas it was inversely associated with LDL-cholesterol and HDL-cholesterol. The characteristics of male and female participants are shown in Supplementary Tables S1 and S2. Although the alcohol dietary pattern was positively associated with body weight, BMI, waist circumference, systolic blood pressure, diastolic blood pressure, AST, *γ*-glutamyl transferase, hepatic steatosis index and TAG in men, there were weak or no associations between these variables and the alcohol dietary pattern in women.

**Table 2.** Characteristics of participants according to the quartile of alcohol dietary pattern score (Mean values and standard deviations, *n* 2171)

	Q1 (*n* 543)		Q2 (*n* 542)		Q3 (*n* 543)		Q4 (*n* 543)		
	Mean	sd	Mean	sd	Mean	sd	Mean	sd	*P* for trend*
Alcohol dietary pattern score	–1·21	0·49	–0·34	0·18	0·26	0·19	1·3	0·59	<0·001
Men (%)	39·2		58·3		70·3		81·6		<0·001
Age (years)	53·7	9·5	53·9	9·6	54·3	9·8	54·2	9·4	0·27
Height (cm)	163·3	7·7	165·4	7·9	166·6	7·5	168·9	7·2	<0·001
Body weight (kg)	58·8	10·8	61·8	11·0	64·0	11·4	67·9	11·4	<0·001
BMI (kg/m^2^)	22·0	3·1	22·5	3·1	23·0	3·3	23·7	3·1	<0·001
Waist circumference (cm)	79·2	9·4	80·6	8·6	82·1	8·8	84·6	9·0	<0·001
SBP (mmHg)	121·6	19·1	124·3	19·1	126·6	20·7	130·5	20·4	<0·001
DBP (mmHg)	75·1	11·3	77·8	12·2	79·6	13·4	81·9	13·3	<0·001
AST (U/l)	22·6	7·6	23·2	8·1	23·8	8·4	26·0	9·6	<0·001
ALT (U/l)	21·4	12·6	22·3	12·8	23·7	13·5	25·2	14·7	<0·001
ALT:AST	0·92	0·29	0·94	0·30	0·97	0·31	0·95	0·29	0·05
*γ*-GTP (U/l)	30·5	34·1	30·6	24·6	38·1	38·6	54·8	60·8	<0·001
HSI	30·6	4·5	30·9	4·4	31·4	4·8	31·8	4·4	<0·001
LDL-cholesterol (mg/dl)	128·8	31·8	126·4	29·7	125·9	29·3	120·8	31·2	<0·001
HDL-cholesterol (mg/dl)	69·5	16·8	67·3	16·4	66·2	17·8	67·1	17·3	0·01
TAG (mg/dl)	87·8	53·4	90·6	59·6	101·3	68·5	123·1	123·3	<0·001
Fasting glucose (mg/dl)	91·1	11·1	93·4	13·9	95·4	15·7	97·0	13·5	<0·001
Fasting insulin (µU/ml)	5·5	4·0	5·4	3·3	5·5	3·3	5·6	3·6	0·34
HOMA-IR	1·3	1·1	1·3	0·9	1·3	0·9	1·4	1·0	0·03
MVPA (min/d)	44·6	46·4	51·4	59·5	52·5	52·1	52·5	47·2	0·01
Marital status (%)	81·6		86·3		86·0		84·2		0·29
Educational status									<0·001
Junior high/high school (%)	3·3		2·4		1·7		0·9		
Junior college and technical college (%)	8·8		9·6		5·0		3·7		
College diploma (%)	87·8		88·0		93·4		95·4		
Household income									<0·001
<3 000 000 JPY (%)	7·9		7·6		6·4		5·9		
3 000 000–5 000 000 JPY (%)	17·1		14·0		12·7		14·0		
5 000 000–7 000 000 JPY (%)	18·0		16·8		17·3		14·4		
7 000 000–10 000 000 JPY (%)	24·7		20·8		22·5		20·6		
>10 000 000 JPY (%)	32·2		40·8		41·1		45·1		
Smoking status									<0·001
Current smoker (%)	3·5		5·0		7·7		12·0		
Former smoker (%)	21·4		29·7		33·1		43·1		
Non-smoker (%)	75·1		65·3		59·1		44·9		
Use of cholesterol-lowering drugs (%)	7·9		8·9		8·8		7·9		1·00
Use of TAG-lowering drugs (%)	1·3		2·0		3·3		5·0		<0·001
Use of antihypertensive drugs (%)	9·9		10·0		13·3		20·4		<0·001
Use of diabetes drugs (%)	1·7		1·8		2·2		3·3		0·06

SBP, systolic blood pressure; DBP, diastolic blood pressure; AST, aspartate aminotransferase; ALT, alanine aminotransferase; *γ*-GTP, *γ*-glutamyl transpeptidase; HSI, hepatic steatosis index; HOMA-IR, homoeostasis model assessment of insulin resistance; MVPA, moderate-to-vigorous physical activity.

* *P* values were obtained from a linear regression analysis for continuous variables and Mantel–Haenszel *χ* ^2^ test for categorical variables.

### Nutrient intake according to the quartile of alcohol dietary pattern score

The nutrient intake according to the quartiles of the alcohol dietary pattern score is shown in Table 3. The alcohol dietary pattern was positively associated with energy intake and energy percentage of protein, alcohol, *n*-3 PUFA, energy-adjusted intake of Na, Mg, P, Fe, Zn, vitamin A, vitamin D, vitamin K, niacin, vitamin B_6_, vitamin B_12_, pantothenic acid and cholesterol. Conversely, the alcohol diet pattern was inversely associated with the energy percentage of fat, carbohydrate, SFA, MUFA, energy-adjusted intake of Ca, Mn, *α*-tocopherol, vitamin B_1_, vitamin C and dietary fibre. The nutrient intake in men and women is shown in Supplementary Tables S3 and S4. The associations of most nutrient intake with the alcohol dietary pattern were similar between men and women. However, although the alcohol dietary pattern was positively associated with the energy percentage of PUFA and *n*-6 PUFA, and energy-adjusted intakes of K, Ca, Cu, *α*-tocopherol, vitamin B_1_ and vitamin B_2_ in women, there were no associations or inverse associations of these variables with alcohol dietary pattern in men.

**Table 3.** Nutrient intake according to the quartile of alcohol dietary pattern score (*n* 2171) (Mean values and standard deviations)

	Q1 (*n* 543)		Q2 (*n* 542)		Q3 (*n* 543)		Q4 (*n* 543)		
	Mean	sd	Mean	sd	Mean	sd	Mean	sd	*P*_for trend_
Energy intake (kcal/d)	1812	499	1925	506	1966	540	2016	539	<0·001
Protein (% energy)	15·0	1·9	15·9	2·5	16·1	3·0	16·4	4·2	<0·001
Fat (% energy)	29·3	4·6	28·7	5·4	28·0	5·8	26·6	6·8	<0·001
Carbohydrate (% energy)	53·9	5·6	51·6	6·7	48·9	7	41·6	8·8	<0·001
Alcohol (% energy)	1·2	2·2	2·9	3·8	5·9	5·7	14·3	9·8	<0·001
Alcohol (g/d)	3·2	6·1	8·1	11·2	16·7	17·9	40·8	30·5	<0·001
SFA (% energy/d)	8·5	1·7	7·8	1·7	7·3	1·7	6·6	1·9	<0·001
MUFA (% energy/d)	10·4	1·8	10·2	2·2	10·0	2·3	9·6	2·7	<0·001
PUFA (% energy/d)	6·6	1·2	6·9	1·4	7·0	1·5	6·8	1·7	0·06
*n*-3 PUFA (% energy/d)	1·2	0·3	1·4	0·3	1·4	0·4	1·5	0·5	<0·001
*n*-6 PUFA (% energy/d)	5·4	1·0	5·5	1·1	5·5	1·2	5·3	1·4	0·19
Na (mg/1000 kcal/d)	2157	369	2266	380	2352	437	2426	532	<0·001
K (mg/1000 kcal/d)	1507	362	1551	410	1531	405	1501	455	0·63
Ca (mg/1000 kcal/d)	326	95	320	99	314	109	303	123	<0·001
Mg (mg/1000 kcal/d)	141	27	148	30	150	32	154	38	<0·001
P (mg/1000 kcal/d)	576	89	601	106	611	125	621	163	<0·001
Fe (mg/1000 kcal/d)	4·4	0·9	4·6	1·1	4·7	1·1	4·7	1·4	<0·001
Zn (mg/1000 kcal/d)	4·3	0·5	4·6	0·6	4·6	0·7	4·5	1	<0·001
Cu (mg/1000 kcal/d)	0·62	0·10	0·64	0·11	0·65	0·11	0·61	0·14	0·31
Mn (mg/1000 kcal/d)	1·8	0·5	1·8	0·5	1·7	0·5	1·5	0·5	<0·001
Vitamin A (μgRAE/1000 kcal/d)*	412	159	452	196	469	207	527	332	<0·001
Vitamin D (µg/1000 kcal/d)	5·9	3·0	7·2	3·6	8·0	4·1	9·2	5·7	<0·001
*α*-Tocopherol (mg/1000 kcal/d)	4·4	0·9	4·4	1·0	4·3	1·0	4·1	1·2	<0·001
Vitamin K (µg/1000 kcal/d)	176	80	194	95	201	94	207	107	<0·001
Vitamin B_1_ (mg/1000 kcal/d)	0·45	0·08	0·46	0·09	0·45	0·10	0·43	0·12	0·004
Vitamin B_2_ (mg/1000 kcal/d)	0·77	0·17	0·78	0·19	0·78	0·19	0·77	0·23	0·70
Niacin (mg/1000 kcal/d)	9·1	1·8	10·1	2·1	10·6	2·3	11·6	3·1	<0·001
Vitamin B_6_ (mg/1000 kcal/d)	0·68	0·15	0·75	0·17	0·77	0·17	0·82	0·20	<0·001
Vitamin B_12_ (µg/1000 kcal/d)	4·2	1·7	5·1	2·0	5·7	2·3	6·7	3·2	<0·001
Folate (µg/1000 kcal/d)	197	61	208	75	206	67	207	78	0·06
Pantothenic acid (mg/1000 kcal/d)	3·7	0·6	3·8	0·7	3·8	0·8	3·8	1·0	0·002
Vitamin C (mg/1000 kcal/d)	73·2	31·2	72·0	32·5	67·0	27·8	60·5	28·2	<0·001
Cholesterol (mg/1000 kcal/d)	207	62	217	82	223	75	228	101	<0·001
Dietary fibre (g/1000 kcal/d)	7·2	1·9	7·3	2·3	7·0	2·1	6·5	2·4	<0·001

RAE, retinol activity equivalent.

* 1 μgRAE = retinol (μg) + *β*-carotene (μg) × 1/12 + *α*-carotene (μg) × 1/24 + *β*-cryptoxanthin (μg) × 1/24 + other provitamin A carotenoids (μg) × 1/24.

### Association between dietary patterns and the prevalence of dyslipidaemia and its components

The prevalence of dyslipidaemia, high LDL-cholesterol, low HDL-cholesterol and high TAG was 45·9 %, 37·0 %, 3·8 % and 16·2 %, respectively. Table 4 shows the OR and 95 % CI for the prevalence of dyslipidaemia and its components according to the quartiles of the alcohol dietary pattern score. A logistic regression model adjusted for potential confounders showed no significant association between alcohol dietary patterns and the prevalence of dyslipidaemia in any model. However, the alcohol dietary pattern was significantly associated with components of dyslipidaemia. It was significantly inversely associated with the prevalence of high LDL-cholesterol (*P*_for trend_ = 0·003), and this inverse association remained significant after adjusting for waist circumference and HOMA-IR in model 2 (*P*_for trend_ = 0·001) and alcohol intake in model 3 (*P*_for trend_ = 0·03). The fully adjusted OR (95 % CI) of the prevalence of high LDL-cholesterol for the lowest to the highest quartile of the alcohol dietary pattern score were 1·00 (reference), 0·83 (0·64, 1·08), 0·84 (0·64, 1·10) and 0·68 (0·49, 0·94), respectively. Furthermore, the alcohol dietary pattern was positively associated with the prevalence of high TAG in model 1 (*P*_for trend_ = 0·02), and multivariate-adjusted OR (95 % CI) of the prevalence of high TAG for the lowest through highest quartile of the alcohol dietary pattern score were 1·00 (reference), 1·03 (0·70, 1·52), 1·12 (0·76, 1·64) and 1·49 (1·03, 2·16), respectively. However, this association was no longer significant in model 2 (*P*_for trend_ = 0·06) and model 3 (*P*_for trend_ = 0·40). In addition, there was no significant association between alcohol dietary patterns and the prevalence of low HDL-cholesterol.

**Table 4.** Prevalence of dyslipidaemia and its components according to the quartile of alcohol dietary pattern score (Odds ratio and 95 % confidence intervals, *n* 2171)

		Q1 (*n* 543)	Q2 (*n* 542)		Q3 (*n* 543)		Q4 (*n* 543)		*P*_for trend_
		OR	OR	95 % CI	OR	95 % CI	OR	95 % CI	
Dyslipidaemia	Number of cases	246	229		255		267		
	Number of cases (per 1000 person)	453	423		470		492		
	Model 1*	1·00 (reference)	0·82	0·64, 1·05	0·92	0·71, 1·19	0·92	0·70, 1·19	0·76
	Model 2†	1·00 (reference)	0·80	0·62, 1·04	0·88	0·68, 1·15	0·83	0·63, 1·09	0·31
	Model 3‡	1·00 (reference)	0·80	0·62, 1·04	0·89	0·68, 1·17	0·86	0·62, 1·18	0·44
High LDL-cholesterol	Number of cases	219	200		205		180		
	Number of cases (per 1000 person)	403	369		378		331		
	Model 1*	1·00 (reference)	0·83	0·65, 1·07	0·83	0·64, 1·08	0·65	0·49, 0·85	0·003
	Model 2†	1·00 (reference)	0·83	0·64, 1·07	0·81	0·62, 1·05	0·60	0·45, 0·79	0·001
	Model 3‡	1·00 (reference)	0·83	0·64, 1·08	0·84	0·64, 1·10	0·68	0·49, 0·94	0·03
Low HDL-cholesterol	Number of cases	25	14		25		19		
	Number of cases (per 1000 person)	46	26		46		35		
	Model 1*	1·00 (reference)	0·59	0·30, 1·16	1·09	0·60, 1·98	0·78	0·40, 1·51	0·84
	Model 2†	1·00 (reference)	0·59	0·29, 1·18	1·11	0·60, 2·05	0·75	0·38, 1·49	0·78
	Model 3‡	1·00 (reference)	0·61	0·30, 1·22	1·25	0·67, 2·35	1·15	0·52, 2·55	0·44
High TAG	Number of cases	57	72		90		132		
	Number of cases (per 1000 person)	105	133		166		243		
	Model 1*	1·00 (reference)	1·03	0·70, 1·52	1·12	0·76, 1·64	1·49	1·03, 2·16	0·02
	Model 2†	1·00 (reference)	1·10	0·73, 1·65	1·17	0·78, 1·75	1·42	0·96, 2·11	0·06
	Model 3‡	1·00 (reference)	1·08	0·72, 1·63	1·11	0·74, 1·67	1·22	0·77, 1·93	0·40

HOMA-IR, homoeostasis model assessment of insulin resistance; MVPA, moderate-to-vigorous physical activity.

* Adjusted for age, sex, marital status, educational status, household income, use of antihypertensive drugs, use of diabetes drugs, smoking status, MVPA, energy intake.

† Additionally adjusted for waist circumference and HOMA-IR.

‡ Additionally adjusted for alcohol intake (g/d).

Since individuals with higher alcohol dietary patterns were likely to use medications, sensitivity analysis excluding those on any medication was performed. After excluding the participants who took any medication (cholesterol-lowering, TAG-lowering, antihypertensive and diabetes drugs), 1737 participants were included in the sensitivity analysis. As shown in Supplementary Table S5, there was no significant change in the OR before and after excluding those on any medication. The association between alcohol dietary pattern and reduced LDL-cholesterol level remained significant after the exclusion. The fully adjusted OR (95 % CI) of the prevalence of high LDL-cholesterol for the lowest to the highest quartile of the alcohol dietary pattern score were 1·00 (reference), 0·77 (0·58, 1·03), 0·81 (0·60, 1·10) and 0·64 (0·44, 0·94), respectively.

Although we focused on an alcohol dietary pattern in the present study, the associations of healthy or traditional Japanese dietary patterns with the prevalence of dyslipidaemia and its components were also analysed, as shown in Supplementary Tables S6 and S7. A healthy dietary pattern was significantly and inversely associated with the prevalence of dyslipidaemia (*P*_for trend_ = 0·04), low HDL-cholesterol (*P*_for trend_ = 0·04) and high TAG (*P*_for trend_ < 0·001) in model 1, and the associations with low HDL-cholesterol and high TAG remained significant in model 3 (*P*_for trend_ = 0·04, low HDL-cholesterol and 0·04 for high TAG, respectively). There was no significant association between healthy dietary patterns and the prevalence of high LDL-cholesterol in any model. The traditional Japanese dietary pattern was not significantly associated with the prevalence of dyslipidaemia, high LDL-cholesterol, low HDL-cholesterol or high TAG in any model.

### Subgroup analysis

Table 5 shows the results of subgroup analysis by sex. We found a significant interaction between sex and the alcohol dietary pattern for the prevalence of dyslipidaemia (*P*_for interaction_ = 0·008 in model 3), while a significant inverse association between alcohol dietary pattern and the prevalence of dyslipidaemia was observed only in women (*P*_for trend_ = 0·01, model 3), not in men. There was no significant interaction between sex and the alcohol dietary pattern for the prevalence of high LDL-cholesterol, and the alcohol dietary pattern was inversely associated with high LDL-cholesterol in both men and women in model 2 (*P*_for trend_ = 0·04, for men and 0·003 for women). This inverse association remained significant after adjusting for alcohol intake in women (*P*_for trend_ = 0·007); however, it was no longer significant in men (*P*_for trend_ = 0·37). We observed an opposing association between the alcohol dietary pattern and the prevalence of high TAG between men and women, although the interaction between sex and the alcohol dietary pattern was not significant (*P*_for interaction_ = 0·07 in model 3). In men, the alcohol dietary pattern was positively and significantly associated with the prevalence of high TAG in model 2 (*P*_for trend_ = 0·02). This association was no longer significant after adjusting for alcohol intake (*P*_for trend_ = 0·12). In contrast, an inverse association between the alcohol dietary pattern and the prevalence of high TAG was observed in women, although it did not reach statistical significance (*P*_for trend_ = 0·14 in model 3).

**Table 5.** Prevalence of dyslipidaemia and its components according to the quartile of alcohol dietary pattern score in men (*n* 1354) and women (Odds ratio and 95 % confidence intervals, *n* 817)

		Q1	Q2		Q3		Q4		
		OR	OR	95 % CI	OR	95 % CI	OR	95 % CI	*P*_for trend_
Men									
*n*		213	316		382		443		
Dyslipidaemia	Number of cases	106	152		201		236		
	Number of cases (per 1000 person)	498	481		526		533		
	Model 1*	1·00 (reference)	0·98	0·69, 1·4	1·18	0·83, 1·66	1·15	0·82, 1·61	0·26
	Model 2†	1·00 (reference)	1·02	0·7, 1·47	1·18	0·83, 1·68	1·09	0·76, 1·55	0·52
	Model 3‡	1·00 (reference)	1·02	0·71, 1·48	1·20	0·83, 1·72	1·13	0·75, 1·69	0·42
High LDL-cholesterol	Number of cases	89	131		157		154		
	Number of cases (per 1000 person)	418	415		411		348		
	Model 1*	1·00 (reference)	1·02	0·71, 1·46	1·01	0·71, 1·43	0·75	0·53, 1·06	0·06
	Model 2†	1·00 (reference)	1·05	0·73, 1·50	1·01	0·71, 1·44	0·73	0·51, 1·04	0·04
	Model 3‡	1·00 (reference)	1·06	0·74, 1·53	1·06	0·74, 1·52	0·83	0·55, 1·24	0·37
Low HDL-cholesterol	Number of cases	10	6		18		12		
	Number of cases (per 1000 person)	47	19		47		27		
	Model 1*	1·00 (reference)	0·40	0·14, 1·14	1·02	0·45, 2·32	0·55	0·23, 1·33	0·54
	Model 2†	1·00 (reference)	0·44	0·15, 1·27	1·04	0·44, 2·42	0·49	0·19, 1·23	0·37
	Model 3‡	1·00 (reference)	0·45	0·16, 1·30	1·11	0·46, 2·64	0·60	0·20, 1·79	0·80
High TAG	Number of cases	35	62		83		127		
	Number of cases (per 1000 person)	164	196		217		287		
	Model 1*	1·00 (reference)	1·26	0·79, 2·01	1·38	0·88, 2·16	1·85	1·20, 2·85	0·003
	Model 2†	1·00 (reference)	1·38	0·84, 2·25	1·47	0·92, 2·36	1·77	1·12, 2·80	0·02
	Model 3‡	1·00 (reference)	1·36	0·83, 2·22	1·41	0·87, 2·28	1·57	0·93, 2·64	0·12
Women									
*n*		330	226		161		100		
Dyslipidaemia	Number of cases	140	77		54		31		
	Number of cases (per 1000 person)	424	341		335		310		
	Model 1*	1·00 (reference)	0·72	0·49, 1·04	0·65	0·43, 0·99	0·64	0·39, 1·07	0·02
	Model 2†	1·00 (reference)	0·64	0·44, 0·95	0·59	0·38, 0·92	0·57	0·34, 0·98	0·007
	Model 3‡	1·00 (reference)	0·64	0·44, 0·95	0·59	0·38, 0·93	0·58	0·31, 1·07	0·01
High LDL-cholesterol	Number of cases	130	69		48		26		
	Number of cases (per 1000 person)	394	305		298		260		
	Model 1*	1·00 (reference)	0·68	0·46, 0·99	0·61	0·39, 0·94	0·57	0·34, 0·98	0·008
	Model 2†	1·00 (reference)	0·62	0·42, 0·92	0·56	0·36, 0·88	0·53	0·30, 0·91	0·003
	Model 3‡	1·00 (reference)	0·62	0·42, 0·92	0·57	0·36, 0·90	0·54	0·29, 1·03	0·007
Low HDL-cholesterol	Number of cases	15	8		7		7		
	Number of cases (per 1000 person)	45	35		43		70		
	Model 1*	1·00 (reference)	0·74	0·30, 1·81	0·90	0·35, 2·32	1·16	0·43, 3·11	0·85
	Model 2†	1·00 (reference)	0·74	0·29, 1·87	1·00	0·38, 2·65	1·16	0·41, 3·31	0·79
	Model 3‡	1·00 (reference)	0·79	0·31, 2·00	1·22	0·45, 3·30	2·23	0·69, 7·27	0·27
High TAG	Number of cases	22	10		7		5		
	Number of cases (per 1000 person)	67	44		43		50		
	Model 1*	1·00 (reference)	0·67	0·30, 1·50	0·72	0·29, 1·76	0·61	0·20, 1·81	0·29
	Model 2†	1·00 (reference)	0·61	0·26, 1·46	0·76	0·29, 1·98	0·58	0·17, 1·98	0·33
	Model 3‡	1·00 (reference)	0·58	0·24, 1·40	0·63	0·23, 1·72	0·32	0·06, 1·64	0·14

HOMA-IR, homoeostasis model assessment of insulin resistance; MVPA, moderate-to-vigorous physical activity.

* Adjusted for age, sex, marital status, educational status, household income, use of antihypertensive drugs, use of diabetes drugs, smoking status, MVPA, energy intake.

† Additionally adjusted for waist circumference and HOMA-IR.

‡ Additionally adjusted for alcohol intake (g/d).

## Discussion

We performed a cross-sectional study to examine the association between alcohol dietary patterns and the prevalence of dyslipidaemia and its components. We subsequently demonstrated that the alcohol dietary pattern characterised by a high intake of alcoholic beverages, liver, chicken and fish was inversely associated with the prevalence of high LDL-cholesterol. Subgroup analysis also showed that the alcohol dietary pattern was inversely associated with the prevalence of dyslipidaemia in women, while it was positively associated with the prevalence of high TAG in men.

In the present study, the prevalence of each dyslipidaemia component differed according to sex (Table 5). In women, the prevalence of low HDL-cholesterol and high TAG was low and most cases of dyslipidaemia had high LDL-cholesterol levels. Therefore, we postulate that the inverse association between the alcohol dietary pattern and the prevalence of dyslipidaemia in women was due to its inverse association with high LDL-cholesterol levels. Conversely, the prevalence of high TAG was higher in men than in women. Although an inverse association between the alcohol dietary pattern and high LDL-cholesterol level was also observed in men, the alcohol dietary pattern was positively associated with the prevalence of high TAG (Table 5). This opposite direction of the associations could explain why the alcohol dietary pattern was not associated with the prevalence of dyslipidaemia in men.

There are several possible reasons why the alcohol dietary pattern was inversely associated with high LDL-cholesterol levels. The first is that alcohol itself may have an LDL-cholesterol-lowering effect; this has been supported by previous studies which reported an inverse association between alcohol intake and LDL-cholesterol levels^(8–10)^. Although the mechanism by which alcohol intake decreases LDL-cholesterol level has not been fully elucidated, it has been suggested that modification of LDL by acetaldehyde, a metabolite of alcohol, enhances the degradation of LDL, thereby reducing LDL-cholesterol levels^(32)^. The second possible reason is that the nutrients consumed with high alcohol intake may have an LDL-cholesterol-lowering effect, which is supported by the inverse association between the alcohol dietary pattern and high LDL-cholesterol levels independent of alcohol intake in this study (Table 4). The factor loadings from the principal component analysis showed that the alcohol dietary pattern in the present study was characterised by a high intake of alcoholic beverages, liver, chicken and variety of fish, and low intake of dairy products, breads, variety of fruits and confectioneries (Table 1). We can assume that several nutrients enriched in these food items potentially affect blood lipid levels. For example, SFA intake in both men and women was lower in individuals with higher alcohol dietary pattern scores (Table 3, Supplementary Table S3 and S4). Intervention studies have shown that substitution of carbohydrates with SFA increases LDL-cholesterol levels^(33)^; it is therefore plausible that a lower SFA intake accompanied by a higher alcohol dietary pattern contributed to the low prevalence of high LDL-cholesterol. Lower carbohydrate intake was also characteristic of individuals with higher alcohol dietary pattern scores (Table 3, Supplementary Table S3 and S4). This seems to contradict previous studies that have demonstrated that a low-carbohydrate diet increases LDL-cholesterol levels^(34,35)^. However, a plant-based low-carbohydrate diet with a low SFA has been shown to decrease LDL-cholesterol levels^(36)^. This suggests that increased intake of SFA, but not decreased intake of carbohydrates, has an LDL-cholesterol-raising effect. In the present study, individuals with higher alcohol dietary pattern scores showed lower intakes of both carbohydrates and SFA, which may contribute to the low prevalence of high LDL-cholesterol. Furthermore, individuals with higher alcohol dietary pattern scores showed a higher intake of micronutrients such as Na, Mg, Fe, vitamin A, vitamin D, vitamin K, niacin, vitamin B_6_ and vitamin B_12_. This raises the hypothesis that these micronutrients synergistically affect LDL-cholesterol levels. Although blood levels of some of these nutrients have been suggested to be associated with blood lipid levels^(37,38)^, the evidence to support this hypothesis remains insufficient.

The subgroup analysis showed a significant association between alcohol dietary patterns and reduced LDL-cholesterol levels after adjusting for alcohol intake only in women, but not in men (Table 5). This result suggests that the association between the alcohol dietary pattern and reduced LDL-cholesterol levels in men was mainly due to the LDL-cholesterol-lowering effect of alcohol, while that in women was due to other dietary factors, but not alcohol intake. The most plausible explanation for this observation could be the difference in nutrient intake across the quartile of the alcohol dietary pattern between men and women. However, the associations of most nutrient intake with the alcohol dietary pattern were similar between men and women (Supplementary Tables S3 and S4). Furthermore, although several nutrient intakes were associated with the alcohol dietary pattern only in women, none of these nutrients were correlated with LDL-cholesterol levels (data not shown). Further studies are needed to identify the nutrient and non-nutrient factors that can explain the sex difference in the association between alcohol dietary patterns and reduced LDL-cholesterol levels.

Unlike the association between the alcohol dietary pattern and high LDL-cholesterol level, a positive association between the alcohol dietary pattern and the prevalence of high TAG was observed (Table 4). This association was no longer significant after adjustment for alcohol intake, suggesting that high alcohol intake contributed to high TAG levels. It is well known that chronic alcohol intake increases the synthesis of very LDL-TAG in the liver^(39)^, which is the main source of circulating TAG in patients with hypertriglyceridaemia. Interestingly, a positive association between the alcohol dietary pattern and high TAG was observed only in men, whereas women showed an inverse association between them, although this association was not statistically significant (Table 5). Consistent with the results of the present study, previous studies have reported that moderate alcohol intake was associated with decreased TAG levels in women^(40,41)^. It has been reported that women have a higher TAG-rich lipoprotein clearance capacity than men^(42)^, which could explain the sex difference in the association between the alcohol dietary pattern and high TAG.

Several studies have reported an association between alcohol dietary patterns and the prevalence of dyslipidaemia or blood lipid levels^(43–46)^. Although the factor loadings characterising alcohol dietary patterns were different among these studies, a positive association between the alcohol dietary pattern score and the prevalence of high TAG or circulating TAG levels was consistently observed. The consistent results among the studies may be due to a TAG-raising effect of alcohol, rather than the diet consumed in conjunction with high alcohol intake, as we suggested in the present study. On the other hand, a few studies have reported an association between alcohol dietary patterns and circulating LDL-cholesterol levels, although results among the studies are inconsistent^(44,46)^. For example, Sauvageot *et al.* identified ‘animal protein and alcohol’ dietary patterns by rank regression analysis in a Western European population and reported a positive association between its score and LDL-cholesterol levels^(44)^. In contrast, Guo *et al.* identified the ‘Izakaya (Japanese Pub)’ dietary pattern by principal component analysis in Japanese men and reported an inverse association between its score and LDL-cholesterol levels^(46)^. There are two possible explanations for the inconsistent results among the studies. The first possible explanation is the difference in the LDL-cholesterol-lowering effect of alcohol intake among populations. A previous study reported a stronger association between serum LDL-cholesterol and alcohol intake in the mutant allele carriers of aldehyde dehydrogenase (*ALDH2*) rs671 polymorphism than that in the homozygous wild-type carriers in the Japanese population^(47)^. This finding suggests that a decreased capacity for acetaldehyde metabolism enhances the LDL-cholesterol-lowering effect of alcohol intake. Considering the allele frequency of *ALDH2* rs671 in a large-scale biobank in Japanese^(48)^, more than 40 % of participants in the present study and the study by Guo et al. are expected to have a mutant allele of *ALDH2* rs671. This possibly contributed to the association between alcohol dietary pattern and reduced LDL-cholesterol levels in these studies. The second possible explanation is the difference in the diet consumed with high alcohol intake among the studies. The ‘animal protein and alcohol’ dietary pattern identified by Sauvageot et al. were characterised by high intakes of lean meat and offal^(44)^, whereas the ‘Izakaya’ dietary pattern identified by Guo *et al.* was characterised by high intakes of fish and seafood^(46)^, like that identified in the present study. These dietary patterns may confound the association between alcohol intake and LDL-cholesterol levels, which may have led to inconsistent results among previous studies, although these studies did not examine whether the associations between dietary patterns and LDL-cholesterol levels were independent of alcohol intake. We showed that the alcohol dietary pattern was inversely associated with high LDL-cholesterol levels, independent of alcohol intake, highlighting the importance of assessing the diet consumed with high alcohol intake for a better understanding of the relationship between alcohol intake and dyslipidaemia.

As expected, a healthy dietary pattern was inversely associated with the prevalence of dyslipidaemia in this study (online Supplementary Table S6). Because the association between the healthy dietary pattern and dyslipidaemia was no longer significant after adjustment for waist circumference and HOMA-IR, this association was possibly mediated by insulin resistance induced by abdominal obesity. Interestingly, a healthy dietary pattern was inversely associated with the prevalence of low HDL-cholesterol and high TAG, but not with the prevalence of high LDL-cholesterol, suggesting that a healthy dietary pattern does not have a preventive effect against high LDL-cholesterol levels. Similar to previous studies^(22,24,43,44)^, the healthy dietary pattern in the present study was characterised by a high intake of vegetables, fruits, soya products and fish. High intake of these food items was associated with a high intake of dietary fibre and micronutrients (data not shown), some of which are potentially associated with decreased levels of LDL-cholesterol. However, a healthy dietary pattern was also associated with a high intake of SFA, which is a major risk factor for high LDL-cholesterol (data not shown). Therefore, the beneficial effects brought about by the healthy dietary pattern may be cancelled by the high intake of SFA. This finding is important, as it suggests that a healthy dietary pattern believed to have beneficial effects on metabolic health is not always associated with a better lipid profile. Although a healthy dietary pattern may have a protective effect against low HDL-cholesterol and high TAG, the alcohol dietary pattern identified in this study would be recommended for individuals predisposed to high LDL-cholesterol if they are free from other metabolic risk factors. Nevertheless, as heavy alcohol consumption is undoubtedly associated with an increased risk of mortality and various chronic diseases, including cancer, it should be noted that drinking too much alcohol is not recommended.

The present study has several limitations. First, its cross-sectional design does not allow inference of causality. Prospective cohort studies or intervention studies are required to elucidate the causal relationship between alcohol dietary patterns and the incidence of dyslipidaemia. Second, the participants of this study were alumni of the same university and their spouses in Japan; therefore, the results may have been affected by selection bias. In fact, the majority of participants in the present study had a household income of more than 10 000 000 JPY, which is much higher than that of the general Japanese population. Given the fact that socio-economic status is reported to be associated with blood lipid and dietary patterns in the Japanese population^(49,50)^, caution should be exercised when generalising our findings to the general Japanese population. Further investigations among representative populations are necessary to generalise our findings to the entire Japanese population and other populations. Third, we did not consider menopausal status in women. Oestrogen has a lipid-lowering effect^(51)^, and postmenopausal women have higher levels of LDL-cholesterol and TAG than premenopausal women^(52)^. Therefore, menopausal status should be included as a covariate.

In conclusion, the present study revealed that the alcohol dietary pattern characterised by a high intake of alcoholic beverages, liver, chicken and fish was associated with the prevalence of dyslipidaemia and its components. This finding provides useful information for the prevention and treatment of dyslipidaemia by modifying the diet.

## Supplementary material

For supplementary material accompanying this paper visit https://doi.org/10.1017/S0007114521002671. click here to view supplementary material
