# Supplementary material for: Association between alcohol dietary pattern and prevalence of dyslipidaemia: WASEDA’S Health Study
Source: Br J Nutr. 2021 Jul 14;127(11):1712–22. doi: 10.1017/S0007114521002671 (PMC9201834; doi:10.1017/S0007114521002671)
Supplement: Supplementary file 1 [file S0007114521002671sup001.docx]

| Table S1. Characteristics of participants according to the quartile of alcohol dietary pattern score in men (n =1354) | | | | | | | | | | |
| --- | --- | --- | --- | --- | --- | --- | --- | --- | --- | --- |
|  | Q1 (n = 213) | | Q2 (n = 316) | | Q3 (n = 382) | | Q4 (n = 443) | |  | |
|  | Mean | SD | Mean | SD | Mean | SD | Mean | SD | P for trend* |  |
| Alcohol dietary pattern score | -1.16 | 0.47 | -0.33 | 0.17 | 0.27 | 0.20 | 1.29 | 0.58 | <0.001 |  |
| Age (year) | 56.7 | 11.1 | 55.7 | 10.2 | 55.3 | 10.1 | 55.0 | 9.6 | 0.06 |  |
| Height (cm) | 170.0 | 5.7 | 170.2 | 5.7 | 169.8 | 5.8 | 171.0 | 5.7 | 0.04 |  |
| Body weight (kg) | 67.0 | 9.1 | 67.2 | 9.2 | 68.1 | 9.7 | 70.9 | 9.9 | <0.001 |  |
| BMI (kg/m^2^) | 23.2 | 2.9 | 23.2 | 2.7 | 23.6 | 3.0 | 24.2 | 2.9 | <0.001 |  |
| Waist circumference (cm) | 83.2 | 8.7 | 82.8 | 7.8 | 84.0 | 8.0 | 86.1 | 8.5 | <0.001 |  |
| SBP (mmHg) | 128.7 | 17.2 | 129.4 | 17.2 | 130.7 | 20.0 | 133.4 | 19.9 | 0.001 |  |
| DBP (mmHg) | 78.8 | 10.4 | 80.9 | 11.7 | 81.9 | 12.8 | 83.6 | 12.9 | <0.001 |  |
| AST (U/L) | 24.1 | 8.9 | 23.9 | 8.5 | 24.6 | 9.0 | 26.8 | 10.0 | <0.001 |  |
| ALT (U/L) | 25.3 | 13.8 | 24.2 | 12.1 | 25.7 | 14.5 | 26.6 | 15.4 | 0.07 |  |
| ALT/AST | 1.03 | 0.32 | 1.00 | 0.30 | 1.02 | 0.32 | 0.98 | 0.30 | 0.05 |  |
| γ-GTP (U/L) | 39.4 | 44.6 | 35.8 | 27.6 | 42.6 | 42.7 | 60.8 | 65.0 | 0.19 |  |
| HSI | 31.6 | 4.7 | 31.2 | 4.4 | 31.9 | 4.7 | 32.2 | 4.4 | 0.02 |  |
| LDL-C (mg/dL) | 129.5 | 31.5 | 129.5 | 27.8 | 127.2 | 27.6 | 121.9 | 30.6 | <0.001 |  |
| HDL-C (mg/dL) | 60.3 | 15.0 | 61.8 | 14.3 | 62.2 | 16.4 | 64.6 | 16.0 | 0.001 |  |
| TG (mg/dL) | 101.8 | 57.9 | 103.0 | 69.5 | 111.2 | 73.4 | 133.2 | 132.6 | <0.001 |  |
| Fasting glucose (mg/dL) | 94.9 | 12.7 | 96.1 | 15.7 | 97.6 | 16.9 | 98.1 | 13.9 | 0.006 |  |
| Fasting insulin (µU/mL) | 6.3 | 4.7 | 5.7 | 3.5 | 5.8 | 3.4 | 5.9 | 3.7 | 0.35 |  |
| HOMA-IR | 1.5 | 1.2 | 1.4 | 1.1 | 1.4 | 1.0 | 1.5 | 1.1 | 0.79 |  |
| MVPA (min/day) | 52.8 | 45.7 | 54.3 | 61.3 | 51.5 | 50.5 | 53.7 | 46.2 | 0.99 |  |
| Marital status (%) | 85.0 | | 90.2 | | 88.5 | | 86.7 | | 0.96 | |
| Education |  | | | | | | | | 0.72 | |
| Junior high/high school (%) | 0.0 | | 0.3 | | 0.8 | | 0.5 | |  | |
| Junior college and technical college (%) | 0.9 | | 1.3 | | 0.8 | | 0.7 | |  | |
| College diploma (%) | 99.1 | | 98.4 | | 98.4 | | 98.9 | |  | |
| Household income |  | | | | | | | | 0.001 | |
| <3,000,000 JPY (%) | 6.1 | | 4.7 | | 7.1 | | 4.1 | |  | |
| 3,000,000–5,000,000 JPY (%) | 19.7 | | 15.8 | | 12.6 | | 14.9 | |  | |
| 5,000,000–7,000,000 JPY (%) | 17.4 | | 17.4 | | 17.8 | | 12.6 | |  | |
| 7,000,000–10,000,000 JPY (%) | 24.9 | | 21.8 | | 19.9 | | 20.8 | |  | |
| >10,000,000 JPY (%) | 31.9 | | 40.2 | | 42.7 | | 47.6 | |  | |
| Smoking status |  | | | | | | | | <0.001 | |
| Current smoker (%) | 6.1 | | 7.0 | | 10.5 | | 12.9 | |  | |
| Former smoker (%) | 38.0 | | 42.1 | | 40.1 | | 47.2 | |  | |
| Non-smoker (%) | 55.9 | | 50.9 | | 49.5 | | 40.0 | |  | |
| Use of cholesterol-lowering drugs (%) | 9.4 | | 11.4 | | 9.9 | | 8.6 | | 0.45 | |
| Use of triglyceride-lowering drugs (%) | 2.3 | | 3.5 | | 4.7 | | 6.1 | | 0.02 | |
| Use of antihypertensive drugs (%) | 15.5 | | 14.2 | | 16.0 | | 23.3 | | 0.003 | |
| Use of diabetes drugs (%) | 3.3 | | 2.8 | | 2.6 | | 3.8 | | 0.61 | |
| Abbreviation: BMI, body mass index; SBP, systolic blood pressure; DBP, diastolic blood pressure; AST, aspartate aminotransferase; ALT, alanine aminotransferase; γ-GTP, γ-glutamyl transpeptidase; HSI, hepatic steatosis index; LDL-C, low-density lipoprotein cholesterol; HDL-C, high-density lipoprotein cholesterol; TG, triglycerides; HOMA-IR, homeostasis model assessment of insulin resistance; MVPA, moderate and vigorous intensity physical activity. *P values was obtained from a linear regression analysis for continuous variables and Mantel-Haenszel χ2 square test for categorical variables. | | | | | | | | | | |

| Table S2. Characteristics of participants according to the quartile of alcohol dietary pattern score in women (n = 817) | | | | | | | | | | | | | | |
| --- | --- | --- | --- | --- | --- | --- | --- | --- | --- | --- | --- | --- | --- | --- |
|  | Q1 (n = 330) | | | Q2 (n = 226) | | | Q3 (n = 161) | | | Q4 (n =100) | | |  |  |
|  | Mean | SD | | Mean | SD | | Mean | SD | | Mean | SD | | P for trend* |  |
| Alcohol dietary pattern score | -1.25 | 0.50 | | -0.36 | 0.18 | | 0.24 | 0.18 | | 1.30 | 0.67 | | <0.001 |  |
| Age (year) | 51.7 | 7.8 | | 51.3 | 8.2 | | 51.9 | 8.6 | | 50.5 | 7.7 | | 0.39 |  |
| Height (cm) | 159.0 | 5.4 | | 158.7 | 5.3 | | 158.9 | 5.3 | | 159.6 | 5.8 | | 0.45 |  |
| Body weight (kg) | 53.5 | 8.1 | | 54.3 | 8.8 | | 54.2 | 8.7 | | 54.8 | 7.7 | | 0.15 |  |
| BMI (kg/m^2^) | 21.2 | 3.0 | | 21.6 | 3.3 | | 21.5 | 3.4 | | 21.5 | 2.8 | | 0.22 |  |
| Waist circumference (cm) | 76.7 | 8.8 | | 77.6 | 8.8 | | 77.4 | 8.9 | | 78.1 | 8.5 | | 0.14 |  |
| SBP (mmHg) | 117.0 | 18.9 | | 117.2 | 19.4 | | 117.0 | 19.3 | | 117.4 | 17.1 | | 0.87 |  |
| DBP (mmHg) | 72.8 | 11.3 | | 73.6 | 11.8 | | 74.0 | 13.1 | | 74.3 | 12.0 | | 0.18 |  |
| AST (U/L) | 21.6 | 6.4 | | 22.1 | 7.6 | | 22.0 | 6.5 | | 22.4 | 6.5 | | 0.25 |  |
| ALT (U/L) | 18.8 | 11.1 | | 19.6 | 13.2 | | 18.8 | 9.4 | | 18.6 | 7.9 | | 0.88 |  |
| ALT/AST | 0.85 | 0.23 | | 0.86 | 0.27 | | 0.84 | 0.23 | | 0.82 | 0.21 | | 0.47 |  |
| γ-GTP (U/L) | 24.8 | 23.4 | | 23.3 | 17.4 | | 27.4 | 23.6 | | 27.9 | 22.7 | | 0.12 |  |
| HSI | 30.0 | 4.2 | | 30.5 | 4.5 | | 30.2 | 4.6 | | 30.1 | 3.7 | | 0.57 |  |
| LDL-C (mg/dL) | 128.4 | 32.0 | | 122.1 | 31.7 | | 123 | 33.1 | | 115.7 | 33.6 | | 0.001 |  |
| HDL-C (mg/dL) | 75.4 | 15.1 | | 75.0 | 16.0 | | 75.5 | 17.6 | | 78.5 | 18.2 | | 0.19 |  |
| TG (mg/dL) | 78.8 | 48.2 | | 73.3 | 35.6 | | 77.6 | 47.8 | | 78.8 | 48.7 | | 0.922 |  |
| Fasting glucose (mg/dL) | 88.5 | 9.0 | | 89.6 | 9.5 | | 90.2 | 10.9 | | 92.3 | 10.6 | | 0.001 |  |
| Fasting insulin (µU/mL) | 4.9 | 3.4 | | 4.9 | 2.9 | | 4.7 | 2.8 | | 4.7 | 3.0 | | 0.46 |  |
| HOMA-IR | 1.1 | 0.9 | | 1.1 | 0.7 | | 1.1 | 0.7 | | 1.1 | 0.8 | | 0.83 |  |
| MVPA (min/day) | 39.4 | 46.1 | | 47.3 | 56.9 | | 54.8 | 55.9 | | 47.5 | 51.2 | | 0.01 |  |
| Marital status (%) | 79.4 | | | 81.0 | | | 80.1 | | | 73.0 | | | 0.34 |  |
| Education |  | |  |  | |  |  | |  |  | |  | 0.61 |  |
| Junior high/high school (%) | 5.5 | | | 5.3 | | | 3.7 | | | 3.0 | | |  |  |
| Junior college and technical college (%) | 13.9 | | | 21.2 | | | 14.9 | | | 17.0 | | |  |  |
| College diploma (%) | 80.6 | | | 73.5 | | | 81.4 | | | 80.0 | | |  |  |
| Household income |  | |  |  | |  |  | |  |  | |  | 0.525 |  |
| <3,000,000 JPY (%) | 9.1 | | | 11.5 | | | 5.0 | | | 14.0 | | |  |  |
| 3,000,000–5,000,000 JPY (%) | 15.5 | | | 11.5 | | | 13.0 | | | 10.0 | | |  |  |
| 5,000,000–7,000,000 JPY (%) | 18.5 | | | 15.9 | | | 16.1 | | | 22.0 | | |  |  |
| 7,000,000–10,000,000 JPY (%) | 24.5 | | | 19.5 | | | 28.6 | | | 20.0 | | |  |  |
| >10,000,000 JPY (%) | 32.4 | | | 41.6 | | | 37.3 | | | 34.0 | | |  |  |
| Smoking status |  | |  |  | |  |  | |  |  | |  | <0.001 |  |
| Current smoker (%) | 1.8 | | | 2.2 | | | 1.2 | | | 8.0 | | |  |  |
| Former smoker (%) | 10.6 | | | 12.4 | | | 16.8 | | | 25.0 | | |  |  |
| Non-smoker (%) | 87.6 | | | 85.4 | | | 82.0 | | | 67.0 | | |  |  |
| Use of cholesterol-lowering drugs (%) | 7.0 | | | 5.3 | | | 6.2 | | | 5.0 | | | 0.49 |  |
| Use of triglyceride-lowering drugs (%) | 0.6 | | | 0.0 | | | 0.0 | | | 0.0 | | | 0.16 |  |
| Use of antihypertensive drugs (%) | 6.4 | | | 4.0 | | | 6.8 | | | 8.0 | | | 0.56 |  |
| Use of diabetes drugs (%) | 0.6 | | | 0.4 | | | 1.2 | | | 1.0 | | | 0.49 |  |
| Abbreviation: BMI, body mass index; SBP, systolic blood pressure; DBP, diastolic blood pressure; AST, aspartate aminotransferase; ALT, alanine aminotransferase; γ-GTP, γ-glutamyl transpeptidase; HSI, hepatic steatosis index; LDL-C, low-density lipoprotein cholesterol; HDL-C, high-density lipoprotein cholesterol; TG, triglycerides; HOMA-IR, homeostasis model assessment of insulin resistance; MVPA, moderate and vigorous intensity physical activity. *P values was obtained from a linear regression analysis for continuous variables and Mantel-Haenszel χ2 square test for categorical variables. | | | | | | | | | | | | | | |

| Table S3. Nutrient intake according to the quartile of alcohol dietary pattern score in men (n =1354) | | | | | | | | | |
| --- | --- | --- | --- | --- | --- | --- | --- | --- | --- |
|  | Q1 (n = 213) | | Q2 (n = 316) | | Q3 (n = 382) | | Q4 (n = 443) | |  |
|  | Mean | SD | Mean | SD | Mean | SD | Mean | SD | P |
| Energy intake (kcal/day) | 1986 | 497 | 2050 | 529 | 2073 | 545 | 2081 | 530 | 0.04 |
| Protein (% energy) | 14.5 | 1.8 | 15.2 | 2.3 | 15.5 | 2.7 | 15.9 | 3.7 | <0.001 |
| Fat (% energy) | 28.9 | 4.9 | 27.8 | 5.3 | 27.1 | 5.6 | 26.0 | 6.1 | <0.001 |
| Carbohydrate (% energy) | 54.2 | 5.9 | 52.4 | 6.8 | 49.8 | 6.8 | 42.1 | 8.6 | <0.001 |
| Alcohol (% energy) | 1.6 | 2.5 | 3.5 | 3.9 | 6.5 | 5.8 | 14.9 | 9.5 | <0.001 |
| Alcohol (g/day) | 4.6 | 7.7 | 10.2 | 12.2 | 19.5 | 19.2 | 43.7 | 30.2 | <0.001 |
| SFA (% energy/day) | 8.3 | 1.9 | 7.6 | 1.7 | 7.1 | 1.7 | 6.5 | 1.8 | <0.001 |
| MUFA (% energy/day) | 10.3 | 2.0 | 9.9 | 2.1 | 9.7 | 2.2 | 9.4 | 2.4 | <0.001 |
| PUFA (% energy/day) | 6.6 | 1.2 | 6.7 | 1.3 | 6.8 | 1.5 | 6.7 | 1.6 | 0.45 |
| n-3 PUFA (% energy/day) | 1.2 | 0.3 | 1.3 | 0.3 | 1.4 | 0.4 | 1.5 | 0.4 | <0.001 |
| n-6 PUFA (% energy/day) | 5.4 | 1.0 | 5.4 | 1.1 | 5.4 | 1.2 | 5.2 | 1.3 | 0.11 |
| Na (mg/1,000 kcal/day) | 2203 | 381 | 2266 | 392 | 2326 | 437 | 2399 | 500 | <0.001 |
| K (mg/1,000 kcal/day) | 1428 | 336 | 1454 | 375 | 1440 | 360 | 1440 | 404 | 0.96 |
| Ca (mg/1,000 kcal/day) | 310 | 89 | 303 | 93 | 292 | 97 | 289 | 112 | 0.006 |
| Mg (mg/1,000 kcal/day) | 136 | 25 | 141 | 27 | 143 | 29 | 149 | 35 | <0.001 |
| P (mg/1,000 kcal/day) | 558 | 81 | 577 | 98 | 583 | 111 | 600 | 143 | <0.001 |
| Fe (mg/1,000 kcal/day) | 4.2 | 0.9 | 4.3 | 1.0 | 4.5 | 1.0 | 4.6 | 1.2 | <0.001 |
| Zn (mg/1,000 kcal/day) | 4.2 | 0.4 | 4.4 | 0.5 | 4.5 | 0.7 | 4.4 | 0.9 | 0.01 |
| Cu (mg/1,000 kcal/day) | 0.60 | 0.10 | 0.62 | 0.10 | 0.63 | 0.11 | 0.60 | 0.14 | 0.59 |
| Mn (mg/1,000 kcal/day) | 1.7 | 0.5 | 1.7 | 0.5 | 1.7 | 0.4 | 1.5 | 0.5 | <0.001 |
| Vitamin A (μgRAE/1,000 kcal/day)* | 391 | 159 | 415 | 179 | 451 | 200 | 499 | 287 | <0.001 |
| Vitamin D (µg/1,000 kcal/day) | 5.6 | 2.6 | 6.6 | 3.1 | 7.3 | 3.6 | 8.8 | 5.2 | <0.001 |
| α-tocopherol (mg/1,000 kcal/day) | 4.2 | 0.8 | 4.2 | 0.9 | 4.1 | 1.0 | 4.0 | 1.1 | 0.002 |
| Vitamin K (µg/1,000 kcal/day) | 162 | 75 | 175 | 83 | 184 | 87 | 199 | 101 | <0.001 |
| Vitamin B_1_ (mg/1,000 kcal/day) | 0.43 | 0.07 | 0.44 | 0.08 | 0.43 | 0.09 | 0.42 | 0.10 | 0.03 |
| Vitamin B_2_ (mg/1,000 kcal/day) | 0.75 | 0.18 | 0.75 | 0.18 | 0.75 | 0.18 | 0.75 | 0.21 | 0.68 |
| Niacin (mg/1,000 kcal/day) | 8.7 | 1.6 | 9.7 | 2.0 | 10.1 | 2.2 | 11.2 | 2.8 | <0.001 |
| Vitamin B_6_ (mg/1,000 kcal/day) | 0.65 | 0.14 | 0.70 | 0.15 | 0.72 | 0.16 | 0.79 | 0.18 | <0.001 |
| Vitamin B_12_ (µg/1,000 kcal/day) | 4.1 | 1.6 | 4.8 | 1.8 | 5.4 | 2.1 | 6.5 | 2.9 | <0.001 |
| Folate (µg/1,000 kcal/day) | 185 | 56 | 191 | 63 | 193 | 58 | 198 | 69 | 0.01 |
| Pantothenic acid (mg/1,000 kcal/day) | 3.6 | 0.6 | 3.7 | 0.7 | 3.7 | 0.7 | 3.7 | 0.9 | 0.01 |
| Vitamin C (mg/1,000 kcal/day) | 68.7 | 30.8 | 65.9 | 29.3 | 62.6 | 24.8 | 58.1 | 26.5 | <0.001 |
| Cholesterol (mg/1,000 kcal/day) | 201 | 67 | 206 | 70 | 210 | 71 | 218 | 87 | 0.003 |
| Dietary fibre (g/1,000 kcal/day) | 6.7 | 1.8 | 6.8 | 2.0 | 6.6 | 1.9 | 6.3 | 2.1 | <0.001 |
| Abbreviation: RAE, retinol activity equivalent *1 μgRAE = retinol (μg) + β-carotene (μg) × 1/12 + α-carotene (μg) × 1/24 + β-cryptoxanthin (μg) × 1/24 + other provitamin A carotenoids (μg) × 1/24 | | | | | | | | | |

| Table S4. Nutrient intake according to the quartile of alcohol dietary pattern score in women (n = 817) | | | | | | | | | |
| --- | --- | --- | --- | --- | --- | --- | --- | --- | --- |
|  | Q1 (n = 330) | | Q2 (n = 226) | | Q3 (n = 161) | | Q4 (n =100) | |  |
|  | Mean | SD | Mean | SD | Mean | SD | Mean | SD | P |
| Energy intake (kcal/day) | 1699 | 468 | 1751 | 414 | 1713 | 436 | 1725 | 481 | 0.61 |
| Protein (% energy) | 15.2 | 1.9 | 16.7 | 2.4 | 17.7 | 3.2 | 18.6 | 5.3 | <0.001 |
| Fat (% energy) | 29.6 | 4.3 | 29.8 | 5.2 | 30.1 | 5.5 | 29.2 | 8.7 | 0.97 |
| Carbohydrate (% energy) | 53.7 | 5.4 | 50.6 | 6.5 | 46.8 | 6.8 | 39.7 | 9.5 | <0.001 |
| Alcohol (% energy) | 1.0 | 1.9 | 2.1 | 3.4 | 4.5 | 5.3 | 11.6 | 10.7 | <0.001 |
| Alcohol (g/day) | 2.3 | 4.7 | 5.1 | 8.8 | 10.3 | 12.0 | 28.2 | 29.0 | <0.001 |
| SFA (% energy/day) | 8.6 | 1.6 | 8.0 | 1.6 | 8.0 | 1.7 | 7.5 | 2.4 | <0.001 |
| MUFA (% energy/day) | 10.4 | 1.7 | 10.6 | 2.1 | 10.7 | 2.1 | 10.5 | 3.5 | 0.32 |
| PUFA (% energy/day) | 6.7 | 1.2 | 7.2 | 1.4 | 7.4 | 1.5 | 7.2 | 2.2 | <0.001 |
| n-3 PUFA (% energy/day) | 1.3 | 0.3 | 1.4 | 0.3 | 1.6 | 0.4 | 1.7 | 0.5 | <0.001 |
| n-6 PUFA (% energy/day) | 5.4 | 1.0 | 5.7 | 1.1 | 5.8 | 1.2 | 5.5 | 1.9 | 0.02 |
| Na (mg/1,000 kcal/day) | 2127 | 358 | 2264 | 363 | 2415 | 431 | 2547 | 646 | <0.001 |
| K (mg/1,000 kcal/day) | 1558 | 368 | 1686 | 420 | 1749 | 424 | 1773 | 561 | <0.001 |
| Ca (mg/1,000 kcal/day) | 336 | 97 | 343 | 103 | 364 | 118 | 362 | 149 | 0.005 |
| Mg (mg/1,000 kcal/day) | 145 | 28 | 157 | 32 | 167 | 32 | 174 | 46 | <0.001 |
| P (mg/1,000 kcal/day) | 588 | 92 | 636 | 107 | 679 | 132 | 710 | 208 | <0.001 |
| Fe (mg/1,000 kcal/day) | 4.5 | 0.9 | 5.0 | 1.1 | 5.3 | 1.2 | 5.4 | 1.7 | <0.001 |
| Zn (mg/1,000 kcal/day) | 4.4 | 0.5 | 4.8 | 0.6 | 5.0 | 0.7 | 5.1 | 1.4 | <0.001 |
| Cu (mg/1,000 kcal/day) | 0.63 | 0.09 | 0.67 | 0.11 | 0.68 | 0.12 | 0.65 | 0.16 | 0.001 |
| Mn (mg/1,000 kcal/day) | 1.8 | 0.5 | 1.8 | 0.6 | 1.7 | 0.5 | 1.5 | 0.4 | <0.001 |
| Vitamin A (μgRAE/1,000 kcal/day)* | 426 | 159 | 505 | 208 | 512 | 217 | 650 | 465 | <0.001 |
| Vitamin D (µg/1,000 kcal/day) | 6.1 | 3.2 | 7.9 | 4.1 | 9.5 | 4.7 | 11.3 | 7.2 | <0.001 |
| α-tocopherol (mg/1,000 kcal/day) | 4.5 | 0.9 | 4.8 | 1.0 | 4.8 | 1.0 | 4.8 | 1.6 | 0.001 |
| Vitamin K (µg/1,000 kcal/day) | 186 | 82 | 222 | 103 | 240 | 97 | 241 | 126 | <0.001 |
| Vitamin B_1_ (mg/1,000 kcal/day) | 0.46 | 0.08 | 0.50 | 0.09 | 0.51 | 0.11 | 0.51 | 0.15 | <0.001 |
| Vitamin B_2_ (mg/1,000 kcal/day) | 0.78 | 0.17 | 0.83 | 0.18 | 0.86 | 0.20 | 0.87 | 0.27 | <0.001 |
| Niacin (mg/1,000 kcal/day) | 9.3 | 1.9 | 10.8 | 2.0 | 11.7 | 2.2 | 13.4 | 3.6 | <0.001 |
| Vitamin B_6_ (mg/1,000 kcal/day) | 0.70 | 0.15 | 0.82 | 0.16 | 0.87 | 0.18 | 0.95 | 0.24 | <0.001 |
| Vitamin B_12_ (µg/1,000 kcal/day) | 4.3 | 1.8 | 5.5 | 2.2 | 6.4 | 2.5 | 7.7 | 4.0 | <0.001 |
| Folate (µg/1,000 kcal/day) | 205 | 64 | 232 | 84 | 236 | 76 | 245 | 100 | <0.001 |
| Pantothenic acid (mg/1,000 kcal/day) | 3.7 | 0.6 | 4.0 | 0.7 | 4.2 | 0.8 | 4.3 | 1.1 | <0.001 |
| Vitamin C (mg/1,000 kcal/day) | 76.2 | 31.2 | 80.4 | 34.9 | 77.5 | 31.6 | 71.0 | 32.9 | 0.39 |
| Cholesterol (mg/1,000 kcal/day) | 211 | 58 | 232 | 94 | 254 | 76 | 273 | 140 | <0.001 |
| Dietary fibre (g/1,000 kcal/day) | 7.5 | 1.9 | 8.0 | 2.4 | 8.0 | 2.3 | 7.6 | 3.0 | 0.09 |
| Abbreviation: RAE, retinol activity equivalent *1 μgRAE = retinol (μg) + β-carotene (μg) × 1/12 + α-carotene (μg) × 1/24 + β-cryptoxanthin (μg) × 1/24 + other provitamin A carotenoids (μg) × 1/24 | | | | | | | | | |

| Table S5. Odds ratios and 95% confidence intervals for the prevalence of dyslipidaemia and its components according to the quartile of alcohol dietary pattern score after excluding those on any medication (n = 1737) | | | | | | |
| --- | --- | --- | --- | --- | --- | --- |
|  |  | Q1 (n = 456) | Q2 (n = 456) | Q3 (n = 431) | Q4 (n = 394) | P for trend |
| Dyslipidaemia | Number of cases | 179 | 160 | 166 | 166 |  |
|  | Number of cases (per 1000 person) | 393 | 351 | 385 | 421 |  |
|  | Model 1* | 1.00 (reference) | 0.78 (0.59–1.03) | 0.86 (0.65–1.15) | 0.98 (0.73–1.32) | 0.95 |
|  | Model 2† | 1.00 (reference) | 0.74 (0.55–0.98) | 0.80 (0.59–1.07) | 0.88 (0.64–1.20) | 0.52 |
|  | Model 3‡ | 1.00 (reference) | 0.73 (0.54–0.97) | 0.76 (0.56–1.03) | 0.75 (0.52–1.08) | 0.12 |
|  |  |  |  |  |  |  |
| High LDL-C | Number of cases | 158 | 139 | 139 | 110 |  |
|  | Number of cases (per 1000 person) | 346 | 305 | 323 | 279 |  |
|  | Model 1* | 1.00 (reference) | 0.79 (0.59–1.06) | 0.84 (0.63–1.13) | 0.68 (0.50–0.94) | 0.04 |
|  | Model 2† | 1.00 (reference) | 0.77 (0.57–1.03) | 0.80 (0.60–1.08) | 0.63 (0.45–0.87) | 0.01 |
|  | Model 3‡ | 1.00 (reference) | 0.77 (0.58–1.03) | 0.81 (0.60–1.10) | 0.64 (0.44–0.94) | 0.04 |
|  |  |  |  |  |  |  |
| Low HDL-C | Number of cases | 9 | 5 | 10 | 8 |  |
|  | Number of cases (per 1000 person) | 20 | 11 | 23 | 20 |  |
|  | Model 1* | 1.00 (reference) | 0.51 (0.23–1.12) | 0.84 (0.41–1.73) | 0.75 (0.34–1.67) | 0.67 |
|  | Model 2† | 1.00 (reference) | 0.46 (0.21–1.04) | 0.76 (0.36–1.61) | 0.60 (0.26–1.38) | 0.37 |
|  | Model 3‡ | 1.00 (reference) | 0.47 (0.21–1.07) | 0.83 (0.39–1.77) | 0.81 (0.31–2.16) | 0.75 |
|  |  |  |  |  |  |  |
| High TG | Number of cases | 37 | 43 | 51 | 74 |  |
|  | Number of cases (per 1000 person) | 81 | 94 | 118 | 188 |  |
|  | Model 1* | 1.00 (reference) | 0.88 (0.54–1.43) | 0.94 (0.59–1.52) | 1.49 (0.94–2.36) | 0.05 |
|  | Model 2† | 1.00 (reference) | 0.84 (0.50–1.40) | 0.92 (0.56–1.51) | 1.32 (0.81–2.15) | 0.15 |
|  | Model 3‡ | 1.00 (reference) | 0.82 (0.49–1.36) | 0.81 (0.49–1.35) | 0.94 (0.53–1.64) | 0.85 |
| Abbreviation: LDL-C, low-density lipoprotein cholesterol; HDL-C, high-density lipoprotein cholesterol; TG, triglycerides; HOMA-IR, homeostasis model assessment of insulin resistance; MVPA, moderate and vigorous intensity physical activity.  *Adjusted for age, sex, marital status, educational status, household income, use of antihypertensive drugs, use of diabetes drugs, smoking status, MVPA, energy intake.  †Additionally adjusted for waist circumference and HOMA-IR.  ‡Additionally adjusted for alcohol intake (g/day). | | | | | | |

| Table S6. Odds ratios and 95% confidence intervals for the prevalence of dyslipidaemia and its components according to the quartile of healthy dietary pattern score (n = 2171) | | | | | | |
| --- | --- | --- | --- | --- | --- | --- |
|  |  | Q1 (n = 543) | Q2 (n = 542) | Q3 (n = 543) | Q4 (n = 543) | P for trend |
| Dyslipidaemia | Number of cases | 264 | 261 | 250 | 222 |  |
|  | Number of cases (per 1000 person) | 486 | 482 | 460 | 409 |  |
|  | Model 1* | 1.00 (reference) | 0.91 (0.71–1.17) | 0.88 (0.68–1.13) | 0.75 (0.57–0.98) | 0.04 |
|  | Model 2† | 1.00 (reference) | 0.91 (0.70–1.17) | 0.90 (0.69–1.18) | 0.83 (0.63–1.10) | 0.22 |
|  | Model 3‡ | 1.00 (reference) | 0.89 (0.68–1.15) | 0.88 (0.67–1.15) | 0.80 (0.60–1.07) | 0.14 |
|  |  |  |  |  |  |  |
| High LDL-C | Number of cases | 193 | 206 | 207 | 198 |  |
|  | Number of cases (per 1000 person) | 355 | 380 | 381 | 365 |  |
|  | Model 1* | 1.00 (reference) | 1.02 (0.79–1.32) | 1.01 (0.78–1.31) | 0.95 (0.72–1.25) | 0.69 |
|  | Model 2† | 1.00 (reference) | 1.03 (0.79–1.33) | 1.04 (0.80–1.36) | 1.02 (0.77–1.36) | 0.85 |
|  | Model 3‡ | 1.00 (reference) | 0.96 (0.74–1.25) | 0.94 (0.72–1.24) | 0.91 (0.68–1.22) | 0.54 |
|  |  |  |  |  |  |  |
| Low HDL-C | Number of cases | 25 | 24 | 20 | 14 |  |
|  | Number of cases (per 1000 person) | 46 | 44 | 37 | 26 |  |
|  | Model 1* | 1.00 (reference) | 0.93 (0.52–1.68) | 0.75 (0.40–1.42) | 0.48 (0.23–1.00) | 0.04 |
|  | Model 2† | 1.00 (reference) | 0.89 (0.48–1.64) | 0.77 (0.40–1.51) | 0.54 (0.25–1.16) | 0.12 |
|  | Model 3‡ | 1.00 (reference) | 0.78 (0.42–1.46) | 0.65 (0.33–1.28) | 0.45 (0.21–0.98) | 0.04 |
|  |  |  |  |  |  |  |
| High TG | Number of cases | 129 | 98 | 78 | 46 |  |
|  | Number of cases (per 1000 person) | 238 | 181 | 144 | 85 |  |
|  | Model 1* | 1.00 (reference) | 0.73 (0.54–1.01) | 0.69 (0.49–0.96) | 0.48 (0.32–0.72) | <0.001 |
|  | Model 2† | 1.00 (reference) | 0.73 (0.52–1.02) | 0.72 (0.50–1.03) | 0.58 (0.38–0.89) | 0.01 |
|  | Model 3‡ | 1.00 (reference) | 0.76 (0.54–1.07) | 0.77 (0.53–1.11) | 0.63 (0.41–0.97) | 0.04 |
| Abbreviation: LDL-C, low-density lipoprotein cholesterol; HDL-C, high-density lipoprotein cholesterol; TG, triglycerides; HOMA-IR, homeostasis model assessment of insulin resistance; MVPA, moderate and vigorous intensity physical activity. *Adjusted for age, sex, marital status, educational status, household income, use of antihypertensive drugs, use of diabetes drugs, smoking status, MVPA, energy intake. †Additionally adjusted for waist circumference and HOMA-IR. ‡Additionally adjusted for alcohol intake (g/day). | | | | | | |

| Table S7. Odds ratios and 95% confidence intervals for the prevalence of dyslipidaemia and its components according to the quartile of traditional Japanese dietary pattern score (n = 2171) | | | | | | |
| --- | --- | --- | --- | --- | --- | --- |
|  |  | Q1 (n = 542) | Q2 (n = 544) | Q3 (n = 543) | Q4 (n = 542) | P for trend |
| Dyslipidemia | Number of cases | 222 | 256 | 244 | 275 |  |
|  | Number of cases (per 1000 person) | 410 | 471 | 449 | 507 |  |
|  | Model 1* | 1.00 (reference) | 1.09 (0.85–1.40) | 0.95 (0.73–1.22) | 1.04 (0.80–1.36) | 0.95 |
|  | Model 2† | 1.00 (reference) | 1.07 (0.83–1.39) | 0.94 (0.72–1.22) | 0.98 (0.75–1.29) | 0.66 |
|  | Model 3‡ | 1.00 (reference) | 1.08 (0.83–1.40) | 0.94 (0.72–1.22) | 0.98 (0.74–1.29) | 0.64 |
|  |  |  |  |  |  |  |
| High LDL-C | Number of cases | 179 | 204 | 199 | 222 |  |
|  | Number of cases (per 1000 person) | 330 | 375 | 366 | 410 |  |
|  | Model 1* | 1.00 (reference) | 1.10 (0.85–1.42) | 1.01 (0.78–1.31) | 1.05 (0.80–1.37) | 0.91 |
|  | Model 2† | 1.00 (reference) | 1.09 (0.84–1.41) | 1.00 (0.77–1.31) | 1.01 (0.77–1.33) | 0.90 |
|  | Model 3‡ | 1.00 (reference) | 1.10 (0.85–1.43) | 1.00 (0.77–1.30) | 1.00 (0.76–1.32) | 0.82 |
|  |  |  |  |  |  |  |
| Low HDL-C | Number of cases | 29 | 17 | 14 | 23 |  |
|  | Number of cases (per 1000 person) | 54 | 31 | 26 | 42 |  |
|  | Model 1* | 1.00 (reference) | 0.61 (0.32–1.13) | 0.50 (0.26–0.99) | 0.87 (0.47–1.61) | 0.50 |
|  | Model 2† | 1.00 (reference) | 0.59 (0.31–1.12) | 0.48 (0.24–0.96) | 0.81 (0.43–1.53) | 0.39 |
|  | Model 3‡ | 1.00 (reference) | 0.59 (0.31–1.12) | 0.48 (0.24–0.95) | 0.80 (0.42–1.51) | 0.36 |
|  |  |  |  |  |  |  |
| High TG | Number of cases | 71 | 101 | 73 | 106 |  |
|  | Number of cases (per 1000 person) | 131 | 186 | 134 | 196 |  |
|  | Model 1* | 1.00 (reference) | 1.18 (0.83–1.69) | 0.77 (0.53–1.13) | 1.22 (0.84–1.78) | 0.76 |
|  | Model 2† | 1.00 (reference) | 1.20 (0.82–1.74) | 0.74 (0.50–1.11) | 1.14 (0.77–1.69) | 0.89 |
|  | Model 3‡ | 1.00 (reference) | 1.18 (0.81–1.72) | 0.75 (0.50–1.11) | 1.15 (0.78–1.71) | 0.96 |
| Abbreviation: LDL-C, low-density lipoprotein cholesterol; HDL-C, high-density lipoprotein cholesterol; TG, triglycerides; HOMA-IR, homeostasis model assessment of insulin resistance; MVPA, moderate and vigorous intensity physical activity. *Adjusted for age, sex, marital status, educational status, household income, use of antihypertensive drugs, use of diabetes drugs, smoking status, MVPA, energy intake. †Additionally adjusted for waist circumference and HOMA-IR. ‡Additionally adjusted for alcohol intake (g/day). | | | | | | |
